# The October Quarterly Meeting of the Illinois State Board of Health, and Our Seaboard Quarantine

**Published:** 1887-12

**Authors:** 


### THE OCTOBER QUARTERLY MEETING OF THE ILLINOIS STATE BOARD OF HEALTH, AND OUR SEABOARD QUARANTINE.

A condensed report of the last meeting of the State Board of Health will be found in another department of this number of the Journal and Examiner. The secretary of the Board has been active in investigating the quarantine stations at the Atlantic seaboard, and in urging their improvement; but if the report, on October 27th, of the special committee appointed by the College of Physicians and Surgeons of New York, to investigate the condition of the quarantine at New York, Philadelphia and Baltimore is correct, he seems to have given more credit to the health authorities of the port of New York than is due them, in his statement that all proper and efficient methods were employed in the case of the steamer Alesia.

The following statements are taken from a leading article in the *Therapeutic Gazette* for November 15th: According to the personal investigation of the committee mentioned, there are no proper provisions at the ports named to prevent the ingress of Asiatic cholera. At New York, the treatment of the passengers of the steamers Alesia and Britannia was as follows:

"Hundreds of men and women, some infected, others not, were for several days crowded together in a single large room. They had at first no bedding at all, and when bedding was received it was simply thrown upon the floor, and during the day piled up in heaps, no care being exercised to see that the same individual had the same blankets on successive nights. There were no proper latrines, no proper protection of the water-supply from infection, and the system of disinfection was so bad that for several days the unfortunate people had no access to any clothes save those on their backs. There was no isolation of the groups of passengers, and no attempt at separation of the possibly infected from the well. There was no medical attendance upon the island during the night. Any person taken with cholera at such a time had to remain without assistance, among the well persons, until the visit of the health officer the next day. Even in the hospital upon an adjacent island, with its ill and dying patients, there was no resident physician, and therefore no proper surveillance of the patients, who apparently, were visited only once in twenty-four hours.

"In the second place, the police supervision was so faulty as practically scarcely to exist, and there can be no doubt that boats passed freely between the infected island and the shore."

This deplorable and dangerous condition of quarantine affairs seems to be due primarily, to an alleged lack of funds, and secondarily to the fact that the quarantine office is a main reliance of one of the political parties of the state of New York for money for campaign expenses. The annual receipts of the office are estimated at from sixty to one hundred thousand dollars. Of this sum, ten thousand is paid to the medical health-officer; seventeen thousand for expenses of the office; and the balance *disappears*. The writer of the article quoted very properly contends that the whole matter of quarantine along the coast is one of general national interest and importance, and that it should be removed from local control, and placed in the hands of the general government.

This subject ought to engage the earnest and active interest of all medical men, since no other class in the community can be considered to be so eminently the guardians of the public health.

